# PDCD6 cooperates with C-Raf to facilitate colorectal cancer progression via Raf/MEK/ERK activation

**DOI:** 10.1186/s13046-020-01632-9

**Published:** 2020-08-03

**Authors:** Xiaojuan Wang, Fan Wu, Han Wang, Xiaoyuan Duan, Rong Huang, Amannisa Tuersuntuoheti, Luying Su, Shida Yan, Yuechao Zhao, Yan Lu, Kai Li, Jinjie Yao, Zhiwen Luo, Lei Guo, Jianmei Liu, Xiao Chen, Yalan Lu, Hanjie Hu, Xingchen Li, Mandula Bao, Xinyu Bi, Boyu Du, Shiying Miao, Jianqiang Cai, Linfang Wang, Haitao Zhou, Jianming Ying, Wei Song, Hong Zhao

**Affiliations:** 1 State Key Laboratory of Medical Molecular Biology, Department of Biochemistry and Molecular Biology, Institute of Basic Medical Sciences, Chinese Academy of Medical Sciences and Peking Union Medical College, Beijing, 100005 China; 2 State Key Laboratory of Membrane Biology, Tsinghua University-Peking University Joint Center for Life Sciences, School of Life Sciences, TsinghuaUniversity, Beijing, 100084 China; 3 Department of Hepatobiliary Surgery and Department of Pathology, State Key Laboratory of Molecular Oncology, National Cancer Center/National Clinical Research Center for Cancer/Cancer Hospital, Chinese Academy of Medical Sciences and Peking Union Medical College, Beijing, 100021 China; 4 Key Laboratory of Gene Editing Screening and R&D of Digestive System Tumor Drugs, Chinese Academy of Medical Sciences and Peking Union Medical College, Beijing, 100021 China; 5 Department of Medical Biology, School of Basic Medical Sciences, Hubei University of Medicine, Shiyan, 442000 China

**Keywords:** PDCD6, Colorectal cancer, Growth, MAPK signaling pathway

## Abstract

**Background:**

Colorectal cancer (CRC) is one of the most common malignancies, and it’s expected that the CRC burden will substantially increase in the next two decades. New biomarkers for targeted treatment and associated molecular mechanism of tumorigenesis remain to be explored. In this study, we investigated whether PDCD6 plays an oncogenic role in colorectal cancer and its underlying mechanism.

**Methods:**

Programmed cell death protein 6 (PDCD6) expression in CRC samples were analyzed by immunohistochemistry and immunofluorescence. The prognosis between PDCD6 and clinical features were analyzed. The roles of PDCD6 in cellular proliferation and tumor growth were measured by using CCK8, colony formation, and tumor xenograft in nude mice. RNA-sequence (RNA-seq), Mass Spectrum (MS), Co-Immunoprecipitation (Co-IP) and Western blot were utilized to investigate the mechanism of tumor progression. Immunohistochemistry (IHC) and quantitative real-time PCR (qRT-PCR) were performed to determine the correlation of PDCD6 and MAPK pathway.

**Results:**

Higher expression levels of PDCD6 in tumor tissues were associated with a poorer prognosis in patients with CRC. Furthermore, PDCD6 increased cell proliferation in vitro and tumor growth in vivo. Mechanistically, RNA-seq showed that PDCD6 could affect the activation of the MAPK signaling pathway. PDCD6 interacted with c-Raf, resulting in the activation of downstream c-Raf/MEK/ERK pathway and the upregulation of core cell proliferation genes such as MYC and JUN.

**Conclusions:**

These findings reveal the oncogenic effect of PDCD6 in CRC by activating c-Raf/MEK/ERK pathway and indicate that PDCD6 might be a potential prognostic indicator and therapeutic target for patients with colorectal cancer.

## Background

Colorectal cancer (CRC) is one of the most common malignancies and is the second-leading cause of cancer-related death worldwide [1]. It is expected that the CRC burden will substantially increase in the next two decades [2]. Despite current progress, many patients with advanced tumors die from this malignancy [3]. To improve the curative effect and prognosis, it is crucial to further explore the molecular mechanism of tumorigenesis and find new biomarkers for targeted treatment.

Mitogen-activated protein kinases (MAPKs) are serine-threonine protein kinases that regulate multiple cellular activities including proliferation, differentiation, and apoptosis [4]. As a major axis of the MAPK pathway, the Raf/MEK/ERK signaling pathway is activated in many human cancers [4, 5]. Raf kinases (A-Raf, B-Raf, and c-Raf) that play indispensable roles in this pathway are regulated by a network of protein-protein interactions and phosphorylation-dephosphorylation events [6, 7]. Generally, Raf kinases are regulated by RAS proteins to activate the Raf/MEK/ERK pathway [8, 9]. However, recent studies have shown that Raf is also regulated by different binding partners, including 14–3-3 proteins, RKIP, KSR, and CNK [10–12]. Some of these binding partners even play important roles in regulating Raf function and tumorigenesis. Cancer treatments that target the c-Raf kinase-inhibitory protein, RKIP, have shown unprecedented response rates [13]. The binding partners of Raf kinases and 14–3-3 proteins negatively regulate the activation of major survival pathways [10, 14]. RUVBL1, which is another c-Raf-binding protein, has been reported to activate the Raf/MEK/ERK pathway and thus promote lung cancer tumorigenesis [15]. These findings indicate that Raf-interacting proteins have an influence on cancer progression.

PDCD6, which is also known as apoptosis-linked gene-2 (ALG-2), encodes a calcium-binding protein that contains five serially repeated EF-hand motifs [16–18]. PDCD6 was originally considered a pro-apoptotic protein that participates in T cell receptor-, Fas-, and glucocorticoid-induced apoptosis [19, 20]. It has also been implicated in diverse physiological processes, including endoplasmic reticulum stress-induced cell death, neuronal apoptosis during organ formation, signal transduction, membrane trafficking, and posttranscriptional control of gene expression [18]. Recent studies have revealed that PDCD6 overexpression can promote the progression of hepatomas, breast, and ovarian cancer, suggesting that PDCD6 might be involved in the maintenance of cellular viability [21–23]. In contrast, downregulating of PDCD6 expression could accelerate gastric cancer, HeLa cells, and glioblastoma cell proliferation [24–26]. These findings suggest PDCD6 has different effects on different tumor types. However, the role of PDCD6 in the pathogenesis of colorectal cancer has not been thoroughly investigated.

In this study, we aimed to identify novel therapeutic targets for CRC. We found that the PDCD6 expression level was elevated in CRC and that PDCD6 overexpression was correlated with poor survival in patients with CRC. The tumor-promoting activity of PDCD6 was characterized by in vitro and in vivo tumorigenesis assays. RNA-Seq and pathway analyses were used to investigate the molecular signaling of PDCD6, and the interaction between PDCD6 and c-Raf was also investigated. Overall, these observations indicated that targeting PDCD6 may provide a new opportunity for treating colorectal cancer.

## Methods

### Cell culture and reagents

HCT116 and HCT15 cells were cultured in Iscove’s Modified Dulbecco’s medium (HyClone) with 10% fetal bovine serum (FBS), while HEK293T cells were cultured in Dulbecco’s modified Eagle medium (HyClone) with 10% FBS. All cell lines were obtained from the Cell Resource Center of Peking Union Medical College. Plasmids were constructed according to a standard cloning technique. PDCD6 was cloned into PCDH-CMV-MCS-EF1-copGFP for overexpression. BAPTM, RAF709, Trametinib, and Oxaliplatin were purchased from Tsbiochem. All compounds were dissolved in DMSO to a final concentration of 10 mmol/ml and stored at − 20 °C.

### Antibodies

Antibodies against PDCD6 were purchased from Proteintech. Antibodies against phospho-c-Raf, c-Raf, phospho-MEK1/2, MEK1/2, phospho-ERK1/2, ERK1/2, phospho-STAT3, Ki67 and cleaved-Caspase3 were purchased from Cell Signaling Technology. Tubulin antibodies were purchased from Santa Cruz Biotechnology, Inc.

### Tissue analysis

A tissue microarray including tumor tissues and their corresponding adjacent normal tissues from 93 cases of CRC was obtained from Shanghai Biochip. A tissue microarray with tumor tissues from 423 cases of CRC was analyzed using paraffin-embedded tumor samples, which were histopathological diagnosed at the Cancer Hospital, Chinese Academy of Medical Sciences. Patient consent and approval from the Institutional Research Ethics Committee were obtained for the use of these clinical materials for research purposes. The clinical information regarding the samples is collected and summarized in Table 1 and Supplementary Table S1. Paraffin-embedded tissue sections (4 mm) were prepared according to standard methods, and the expression of PDCD6 (1:300 dilution) was detected using immunoperoxidase. Slides were assessed by pathologists who were blinded to the experimental results and patient outcomes. The PDCD6 expression was evaluated by an immunostaining score, which was calculated as the sum of the proportion and intensity of the stained tumor cells. Briefly, a proportion score, which represented the estimated proportion of positively stained tumor cells (0, none; 1, 0 ~ 25%; 2, 25 ~ 50%; 3, 50 ~ 75%; and 4, 75 ~ 100%.), was first assigned. Next, an intensity score, which indicated the average intensity of positively stained tumor cells (0, none; 1, weak; 2, intermediate; and 3, strong) was obtained. The proportion and intensity scores were then added to obtain a total score, which ranged from 0 to 12.

**Table 1:** Correlation of the expression of PDCD6 in colorectal cancer with clinicopathologic parameters

Clinicopathologic parameters	N	PDCD6		*P*-value
		Weak (IHC Score ≤ 5)	Strong (IHC Score > 5)	
Gender				0.7875
Male	238	180	58	
Female	185	142	43	
Age				0.9413
< 60	229	174	55	
≥ 60	194	148	46	
Differentiation				0.601
Low	47	33	14	
Moderate	354	272	82	
High	22	17	5	
Tumor size (cm)				0.2589
< 5	282	210	72	
≥ 5	141	112	29	
pTNM				0.0020**
I	31	23	8	
II	187	154	33	
III	182	134	48	
IV	23	11	12	
pT				0.0305*
T1	1	1	0	
T2	47	37	10	
T3	334	261	73	
T4	41	23	17	
pN				0.2374
N0	236	187	49	
N1	132	93	39	
N2	53	40	13	
N3	2	2	0	
KRAS				0.6311
Negative	360	272	88	
Positive	63	50	13	
BRAF				0.5766
Negative	419	318	101	
Positive	4	4	0	

*pTNM* pathological tumor-node-metastasis; *pT* pathological tumor; *pN* pathological node. The significance of PDCD6 expression in clinicopathologic parameters was analyzed by Pearson’s chi-squared test. If the expected counts were less than 5, Fisher’s exact test was used to analyze the statistics (*, *p* < 0.05; **, *p* < 0.01)

### Immunofluorescence staining

Dissect tissue as fast as possible, then immerse in fixative (Servicebio. G1101) immediately. Trim tissue sample appropriately after fixation (at least 24 h). Immerse sample in 15% sugar (Sinopharm. 57–50-1) solution at 4 °C until sink down to the bottom, then transfer to 30% sugar solution at 4 °C. Take out a tissue sample from 30% sugar solution and remove the redundant solution. Mount sample in OCT compound (Sakura. 4583) and freeze at − 20 °C to − 80 °C. Cut 8-10 μm sections in cryostat and mount on histological slides. The tissues were washed with PBS. The tissues were fixed with 4% formaldehyde and permeabilized with PBS containing 0.3% Triton X-100. The tissues were blocked with 5% BSA at room temperature for 1 h and then incubated with PDCD6 at 4 °C overnight. Alexa Flour 488 FITC-conjugated secondary antibodies were added and incubated for 30 min at 37 °C.

Live cells were washed with PBS. The cells were fixed with 4% formaldehyde and permeabilized with PBS containing 0.3% Triton X-100. The cells were blocked with 5% BSA at room temperature for 1 h and then incubated with PDCD6 and c-Raf antibodies at 4 °C overnight, respectively. Alexa Flour 488/594 FITC-conjugated secondary antibodies were added and incubated for 30 min at 37 °C. The slides were stained with DAPI, mounted, and observed under a microscope. As a negative control, the specific primary antibodies were replaced with a control mouse or rabbit IgG antibody.

### Lentivirus-mediated transduction

For the knockdown and overexpression of human PDCD6, the PDCD6-specific shRNAs and the PDCD6 sequence were cloned into the vectors pLKO.1 puro and pCDH-CMV-MCS-EF1-copGFP, respectively. The pCMV-VSV-G and psPAX2 were used as helper plasmids to produce the lentivirus. The sequences are shown in Supplementary Table S2. The virus was harvested 48 h after transfecting in HEK293T cells. The target cells were infected by the viral supernatants, which were diluted fourfold in a fresh medium. To isolate the cells that expressed GFP, the cells were dissociated into a single-cell suspension using trypsin and aggregates were removed by a 40-μm cell strainer. FACS was performed using a BD Aria II sorter, which was gated for a moderate level of GFP expression.

### qRT-PCR

Total RNA was isolated from the different cell lines using TRIzol reagent (Invitrogen) according to the manufacturer’s instructions. Equal amounts of RNA were reverse transcribed into cDNA using a Revert Aid First Strand cDNA synthesis kit (Thermo Scientific) according to the manufacturer’s instructions. Quantitative PCR was performed using an ABI Step One Plus system. The PCR reactions were carried out in 10 μL reactions using SYBR Green PCR master mix (Invitrogen) and 0.5 μM specific primers. The primers used for PCR are shown in Supplementary Table S3.

### Cell proliferation assay and colony formation assays

Cell proliferation was assessed using a CCK-8 assay (Dojindo Molecular Technologies). Briefly, HCT116 and HCT15 cells were seeded in 96-well plates. Ten microliters of CCK-8 solution was added to each well containing 100 μL culture medium and incubated for 2 h at 37 °C. The absorbance was measured at a wavelength of 450 nm using an ELISA plate reader. For the cell proliferation assays, cell growth was analyzed once per day for 6 days. For the colony formation assays, 200 cells per well were seeded in six-well plates and cultured at 37 °C for 2 weeks. At the end of the incubation, the cells were fixed with 1% paraformaldehyde for 30 min and stained with 0.1% (w/v) crystal violet for 30 min. Cell colonies were counted.

### In vivo tumorigenesis assays

Animal experiments were performed with the approval of the Peking Union Medical College Animal Care and Use Committees. Five million tumor cells were resuspended in 0.2 ml phosphate-buffered saline and inoculated into the flanks of 6-week old male athymic nude mice (6 mice in each group). Tumor growth was monitored every 3 days by measuring tumor diameters. Tumor width (W) and length (L) were measured, and the tumor volume was calculated using the following formula: volume = (W × L)^2^/2. The mice were sacrificed at 25 days after inoculation. The tumors were removed, photographed, and weighed and the average weights of the tumors were obtained (*P < 0.05).

### RNA-seq and bioinformatics analysis

Total RNA was isolated, as previously described. Novogene Technology Co., Ltd. (Tianjin, China) prepared the libraries and performed the sequencing. RNA-seq was performed to detect the mRNA expression profiles of PDCD6 knockdown colorectal cancer cells using HiSeq3000 (Illumina). LifeScope v2.5.1 was used to align the reads to the genome, generate raw counts corresponding to each known gene, and calculate the FPKM (fragments per kilobase million) values. Differentially expressed genes with a fold change > 2 were selected, and gene ontology (GO) analysis was used for pathway enrichment using Cytoscape (ClueGo) with a *P*-value < 0.05.

### Mass spectrometry (MS)

The HCT116 cells were lysed in lysis buffer (50 mM Tris-HCl (pH 7.4), 100 mM NaCl, 0.5 mM Ca^2+^, 0.5% NP-40 and protease inhibitor cocktails) and subjected to affinity purification with anti-PDCD6 antibody. The purified protein complex was resolved on SDS-PAGE and Coomassie brilliant blue stained. The gel bands of interest were excised from the gel. Peptides were analyzed by Thermo Scientific Q Exactive mass spectrometer.

The MS/MS spectra from each LC-MS/MS run were searched against the raf1.fasta from UniProt using an in-house Proteome Discoverer (Version PD1.4, Thermo-Fisher Scientific, USA). The false discovery rate (FDR) was also set to 0.01 for protein identifications.

### Co-IP assay

Immunoprecipitation assays were performed, as previously described [27]. For coimmunoprecipitation assays, HCT116 cells were harvested and lysed in lysis buffer (50 mM Tris-HCl (pH 7.4), 100 mM NaCl, 0.5 mM Ca^2+^, 0.5% NP-40 and protease inhibitor cocktails). Immunocomplexes were solubilized in 5 × SDS loading buffer and immunoblotted with the indicated antibodies.

### Immunohistochemical (IHC) staining

5 μm longitudinal sections of the paraffin-embedded femurs were kept at 60 °C for 24 h in the oven and then followed by deparaffinized with xylene and hydrated with an ethanol gradient (100–70%). After successively incubating with antigen retrieval solution (Shanghai Shunbai Biotechnology Company; Shanghai, China) and 3% H2O2 for 30 min, the slides were rinsed with water and incubated with the primary antibody (IGF-1 (1: 50)) overnight at 4 °C. For negative controls, the primary antibody was replaced by nonimmunized serum. The next day, the slides were rinsed and incubated with the corresponding secondary antibody (Beijing Biosynthesis Biotechnology Co. Ltd.; Beijing, China) for 30 min followed by 3,3′-diaminobenzidine (DAB) and hematoxylin staining, respectively.

### Hematoxylin-eosin (HE) staining

After deparaffinization and rehydration, 5 μm longitudinal sections were stained with hematoxylin solution for 5 min followed by 5 dips in 1% acid ethanol (1% HCl in 70% ethanol) and then rinsed in distilled water. Then the sections were stained with eosin solution for 3 min and followed by dehydration with graded alcohol and clearing in xylene.

### Statistical analysis

Comparisons between PDCD6 expression in CRC tissues and adjacent normal tissues were analyzed by the Wilcoxon signed-rank test. Associations of the relationship between PDCD6 expression and the clinicopathological parameters of 93 CRC patients were analyzed using the χ^2^ test . We estimated the expression of PDCD6 for each patient and categorized the patients into PDCD6-low and PDCD6-high groups according to the optimal cutoff (5) obtained in Xtile software. Disease-free survival was analyzed by the Kaplan-Meier method and tested by the log-rank test. For the cytological studies, the data are presented as means ± SDs of independent experiments which were repeated three times. The significance of differences between experimental groups was analyzed using a Student’s *t-*test. *P* < 0.05 was considered statistically significant. All the analyses were performed by GraphPad Prism 7.0. software.

## Results

### PDCD6 is overexpressed in colorectal tumors and its overexpression is associated with a poor prognosis in patients with colorectal tumors

**Fig. 1:**
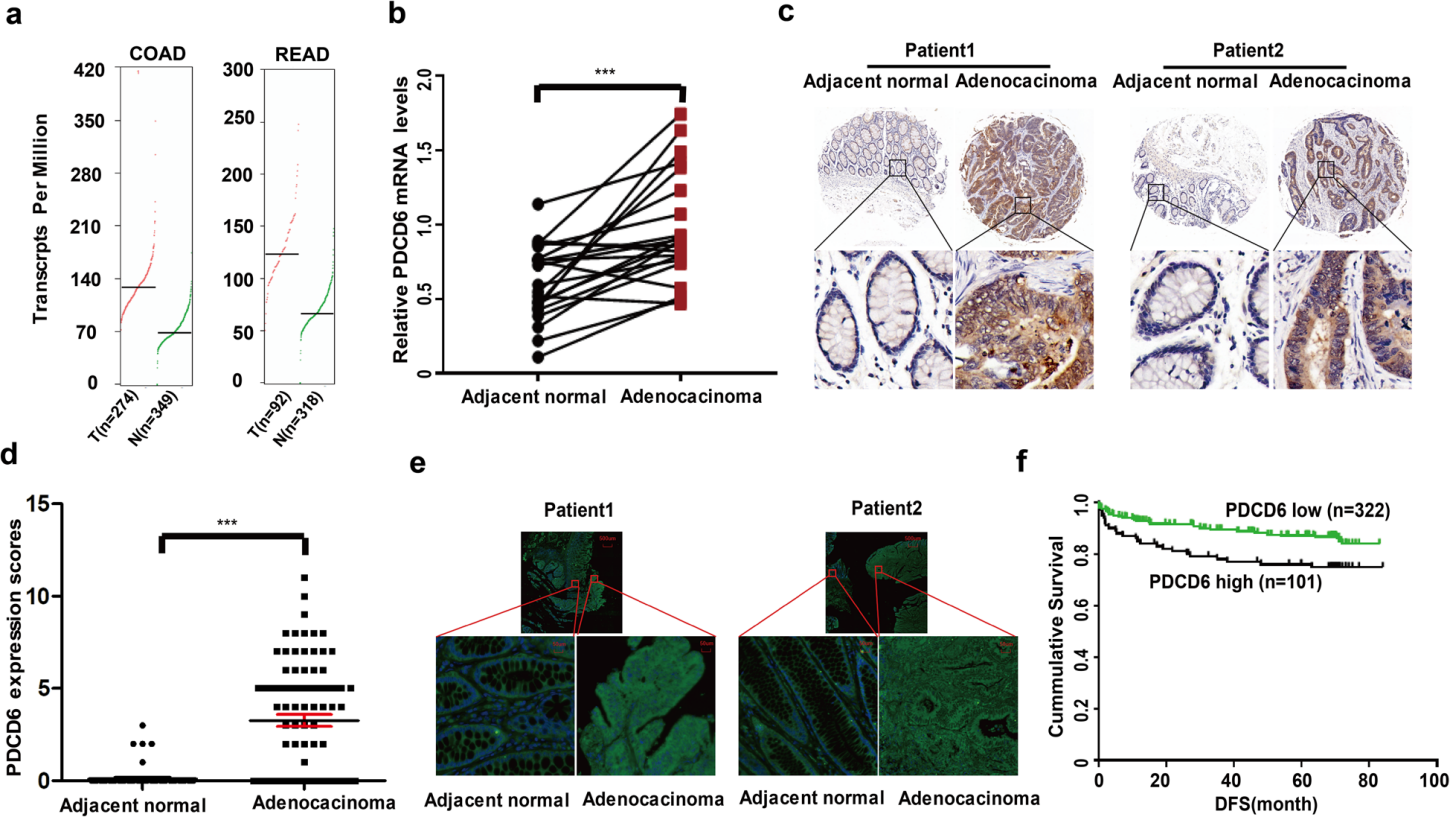
PDCD6 is overexpressed in colorectal tumors and its overexpression is associated with poor prognosis in patients with colorectal tumors. **a**. GEPIA database analysis of PDCD6 expression in 623 patients with CORD and 410 patients with READ. **b**. Relative mRNA levels of PDCD6 in CRC patients was analyzed by qRT-PCR. The data are presented as means ±SDs; *N* = 22, and ***, *P* < 0.001. **c**. Representative immunohistochemical staining of PDCD6 on tissue microarrays containing CRC tissues and adjacent normal tissues. **d**. Tissue microarray data analysis of PDCD6 expression in 93 patients with CRC. The data are presented as the means ±SDs; *N* = 93; and ***, *P* < 0.001. **e**. Immunofluorescence staining of PDCD6 in patients with CRC tissues and adjacent normal tissues. **f**. Kaplan-Meier survival analysis of the correlation between PDCD6 expression and disease-free survival (DFS) in 423 patients with CRC. *N* = 423; DFS: *P* = 0.0103

To investigate the role of PDCD6 in CRC progression, we analyzed the PDCD6 mRNA transcripts of different patient samples in the GEPIA database and found that PDCD6 was highly expressed in multiple kinds of cancer, including breast invasive carcinoma (BRCA), diffuse large B-cell lymphoma (DLBC), esophageal carcinoma (ESCA), colon adenocarcinoma (COAD) and rectal adenocarcinoma (READ) (Fig. 1a and Supplementary Fig.S1). We validated the results in CRC and adjacent normal tissues from 22 patients using qRT-PCR and obtained the same results (Fig. 1b). We further compared the expression levels of PDCD6 in CRC and adjacent normal tissues using tissue microarrays containing 93 CRC samples (Supplementary Table S1). Immunohistochemical staining of representative samples showed that PDCD6 was detectable in most CRC tissues, but it was weakly detected in the adjacent normal tissues (Fig. 1c). Statistically, PDCD6 expression was significantly upregulated in CRC tissues compared with adjacent normal tissues (Fig. 1d). Furthermore, immunofluorescence analysis also showed higher expression levels of PDCD6 in tumor tissues than in normal tissues of the same representative CRC samples (Fig. 1e). Next, we analyzed the correlation between the PDCD6 expression and the clinicopathological parameters of 423 patients with CRCs (Table 1). Statistical analysis showed that PDCD6 expression in colorectal cancer tissues was positively correlated with the pathological tumor-node-metastasis (pTNM) stages and pathological tumor (pT) stages, respectively. No significant correlation was found between PDCD6 expression and other parameters including the pathological node (pN) classification, gender, age, differentiation degree, tumor size, and the mutation status of KRAS and BRAF. Survival analysis showed that a high PDCD6 expression level was significantly correlated with poor disease-free survival (DFS) (Fig. 1f). These data suggest that PDCD6 has the potential clinical value as a predictive biomarker for disease diagnosis in CRC and the patients with CRC benefit from the elimination of PDCD6.

### PDCD6 depletion decreases tumor cell growth in vitro and in vivo

**Fig. 2:**
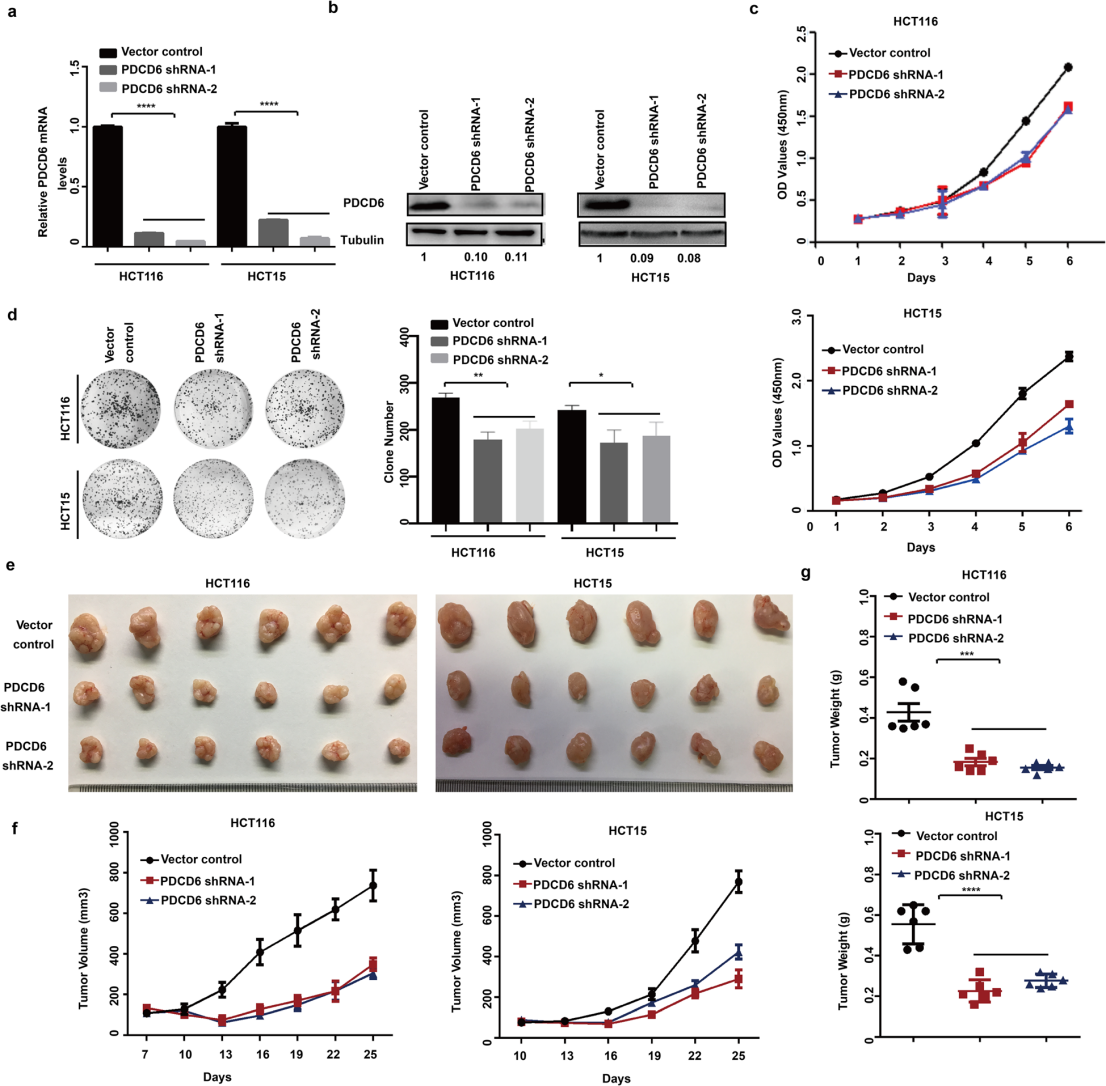
PDCD6 inactivation decreases tumor cell growth in vitro *and* in vivo*.***a**. RT-PCR expression analysis of PDCD6 mRNA in HCT116 and HCT15 cell lines. The data are presented as the means ± SDs, *N* = 3; and ***, *P* < 0.0001. **b**. Immunoblotting of PDCD6 protein expression in HCT116 and HCT15 cell lines. **c**. Cell proliferation assays. The samples were assayed in triplicate. Each datapoint is the mean value of three independent samples. **d**. Colony formation assays. The representative images and bar graphs are from three independent experiments. The data are presented as the means ± SDs, *N* = 3; *P < 0.05; and **, *P* < 0.01. **e**. Images of xenograft tumors. The tumors were removed and photographed. *N* = 6. **f**. Growth curves of xenograft tumors. Tumor volumes were monitored every 3 days by measuring tumor diameters. The data are presented as the means ± SDs; N = 6. **g**. Weights of xenograft tumors. The tumors were removed, photographed, and weighed. The bar graphs show the means ± SDs; *N* = 6; ***, *P* < 0.001; and ****, *P* < 0.0001

To investigate the PDCD6 function in colorectal cancer, we further generated stable PDCD6-knockdown (PDCD6-KD) HCT116 and HCT15 cell lines. As shown in Fig. 2a and b, PDCD6 expression was strongly inhibited at both the RNA and protein levels. Proliferation assays and colony formation assays showed that both cell proliferation and the colony formation ability were decreased in PDCD6-KD HCT116 and HCT15 cells compared with cells expressing the vector control (Fig. 2c and d). We injected HCT116 and HCT15 stable cells into nude mice to explore the effect of PDCD6-KD on tumor growth in vivo. The tumor sizes of the PDCD6-KD group xenografts were markedly smaller than those in the control group (Fig. 2e). Tumors originating from the vector control cells reached over 800mm^3^ within 25 days, whereas the tumors originating from the PDCD6-KD cells were 400mm^3^ at the end of the experiment (Fig. 2f). Accordingly, the weights of tumors originating from PDCD6-KD HCT116 and HCT115 cells decreased more than 2- and 3-fold respectively compared with those originating from the control cells (Fig. 2g). Collectively, these in vitro and in vivo experiments demonstrate that PDCD6 depletion significantly inhibits tumor cell growth.

### PDCD6 overexpressed promotes colorectal cancer growth

**Fig. 3:**
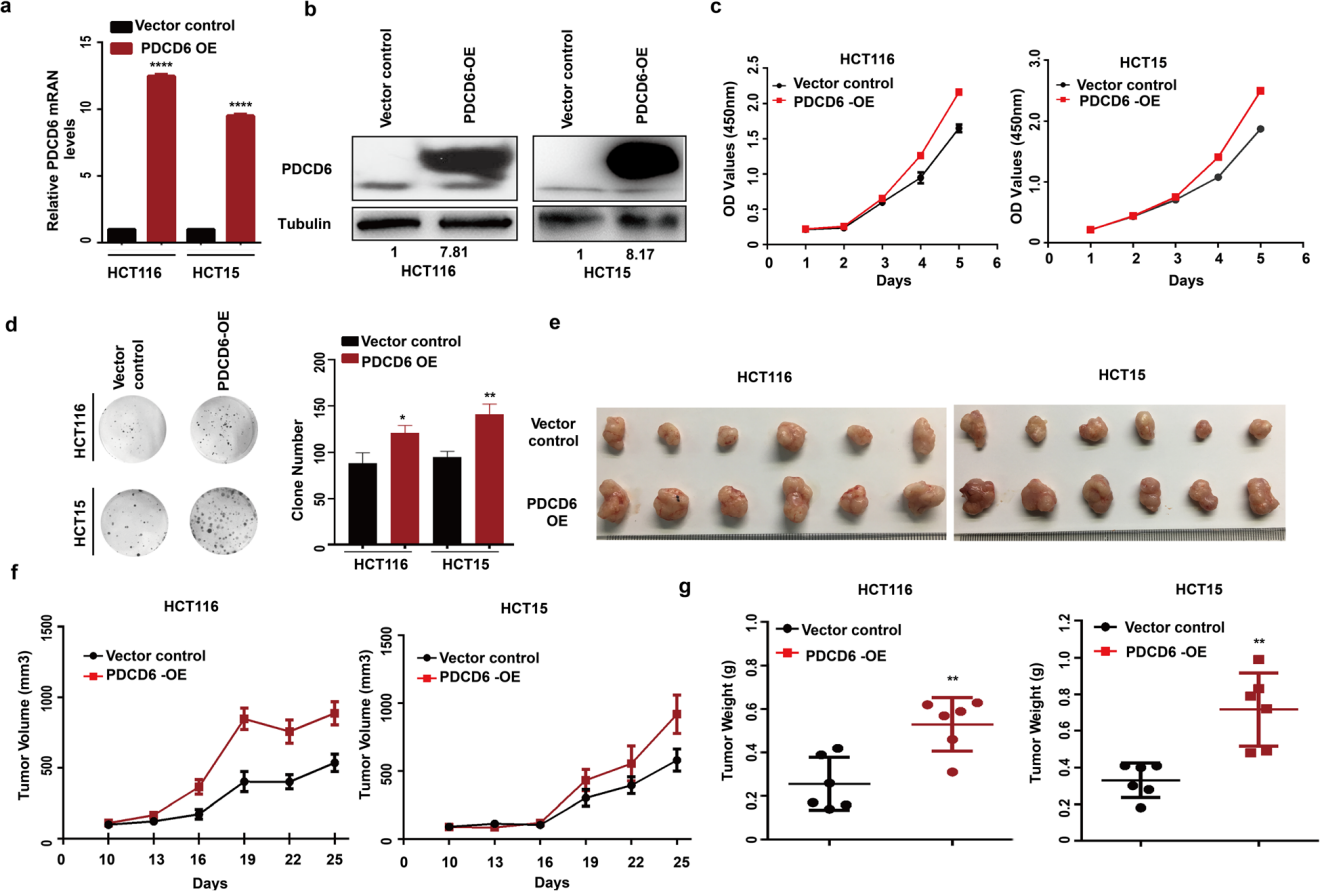
PDCD6 overexpression promotes colorectal cancer growth. **a**. RT-PCR expression analysis of PDCD6 mRNA in HCT116 and HCT15 cell lines. The data are presented as the means ± SDs; *N* = 3; and ***, *P* < 0.0001. **b**. Immunoblotting of PDCD6 protein expression in HCT116 and HCT15 cell lines. **c**. Cell proliferation assays. The samples were assayed in triplicate. Each datapoint is the mean value of three independent samples. **d**. Colony formation assays. The representative images and bar graphs are from three independent experiments. The data are presented as the means ± SDs; N = 3; *, *P* < 0.05; and **, *P* < 0.01. **e**. Images of xenograft tumors. The tumors were removed and photographed. **f**. Growth curves of xenograft tumors. Tumor volumes were monitored every 3 days by measuring tumor diameters. The data are presented as the means ± SDs; N = 6. **g**. Weights of xenograft tumors. The tumors were removed, photographed, and weighed the bar graphs show the means ± SDs; *N* = 6; and **, *P* < 0.01

Furthermore, we further generated stable PDCD6-overexpressing (PDCD6-OE) HCT116 and HCT15 stable cells (Fig. 3a and b). Compared with the control group, the PDCD6 overexpression significantly promoted the proliferation and colony-forming ability of the HCT116 and HCT15 cells (Fig. 3c and d). We injected stable HCT116 and HCT15 stable cells into nude mice to explore the effect of PDCD6-OE on tumor growth in vivo. Tumors originating from the wild-type HCT116 and HCT15 cells reached over 700mm^3^ within 25 days, whereas the tumors originating from the PDCD6-OE cells were 900mm^3^ at the end of the experiment. The tumor sizes and weights of the PDCD6-OE group xenografts were significantly greater than those of the vector control xenografts (Fig. 3e-g). Overall, these data reveal that PDCD6 overexpression promotes tumor cell growth.

### PDCD6 depletion attenuates MAPK signaling pathway activity

**Fig. 4:**
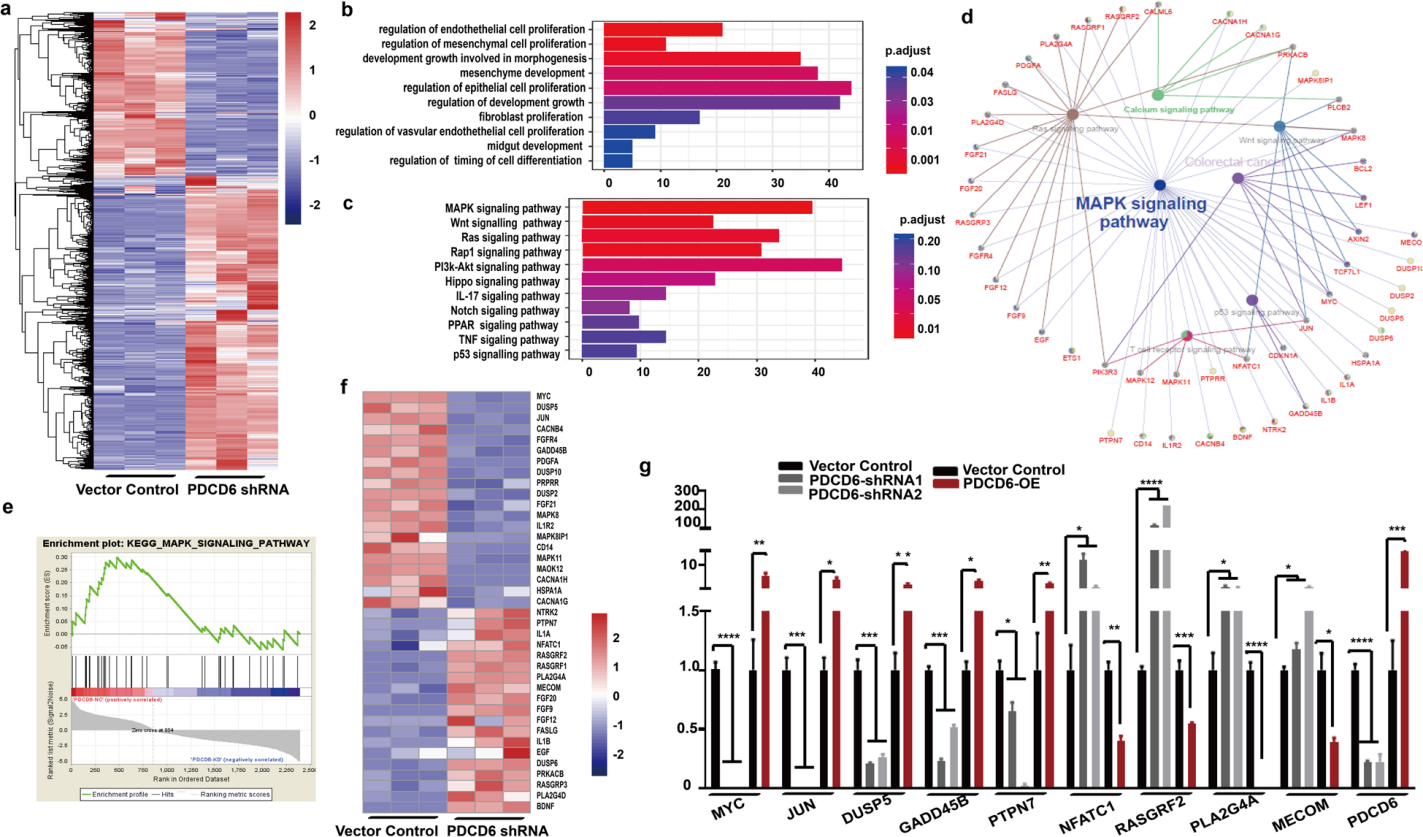
Suppression of PDCD6 expression attenuates MAPK signaling pathway activity. **a**. A heatmap of differentially expressed genes in PDCD6-knockdown and control HCT15 cells that were generated using R software. The log_2_ values were calculated for each sample by normalizing the data to the number of reads alone (*P* < 0.05). **b**. The functional category based on gene ontology (GO) term enrichment. **c**. The signaling pathway based on KEGG enrichment analysis of signal transduction pathway sub-categories. **d**. Functionally grouped networks based on KEGG pathway analysis. The genes are presented for the PDCD6-KD and control cells. **e**. Gene set enrichment analysis (GSEA) of genes involved in cell proliferation in HCT15 PDCD6 knock-down cells with the gene sets corresponding to the MAPK signaling pathway in the KEGG database (NES Score = 0.05 and FDR = 0.10). **f**. A heatmap, which was generated using R software, of MAPK signaling pathway-related genes that were differentially expressed in HCT15 vector control and PDCD6 knock-down cells using R software. The log_2_ values were calculated for each sample by normalizing the data to the number of reads alone (*p* < 0.05 and FDR < 0.05). **g**. Quantitative real-time PCR analysis of the relative mRNA expression of MYC, JUN, DUSP5, GADD45B, PTPN7, NFATC1, RASGFR2, PLA2G4A, MECOM and PDCD6 in control and PDCD6-knockdown HCT15 cells normalized to actin expression (means± SDs, t-test, and *, *p* < 0.05). The experiments were repeated three times

Next, we conducted RNA-Seq analyses to explore the underlying molecular mechanism by which PDCD6 contributes to CRC growth. We analyzed differentially expressed genes and constructed a heatmap. A total of 2416 genes, including 1560 upregulated genes and 856 downregulated genes, were differentially expressed in silenced HCT15 cells and their control counterpart (Fig. 4a). Functional categories based on gene ontology (GO) term enrichment were conducted to explore the possible role of PDCD6. The functions of cell proliferation and growth were significantly enriched (Fig. 4b). We applied KEGG analysis to explore the role of development-related signaling pathways and found that the MAPK signaling pathway was a dominant component in the enriched pathways (Fig. 4c). To further investigate the functionally grouped networks in CRC, the ClueGO and the CluePedia plugins of in Cytoscape were used to identify the enriched pathways involved in tumorigenesis and to observe a functionally grouped network between the PDCD6-KD and the control groups. The results showed that there was a close correlation between the MAPK pathway and CRC (Fig. 4d). Gene set enrichment analysis (GSEA) indicated that the expressions of downstream target genes in the MAPK signaling pathways were significantly suppressed (Fig. 4e). Moreover, the differential gene expression signatures of the MAPK pathway were also recapitulated in a heatmap (Fig. 4f). The qRT-PCR analysis was performed using PDCD6-KD and PDCD6-OE HCT15 cells to analyze the transcription levels of 9 important genes, which are known to take part in tumor progression. The transcription levels of the 9 genes were also consistent with the heatmap data, in which genes were known to take part in tumor growth [28–33] (Fig. 4g). Overall, these results show that PDCD6 affects the MAPK signaling pathway.

### PDCD6 physically interacts with c-Raf and activates the MAPK signaling pathway in CRC

**Fig. 5:**
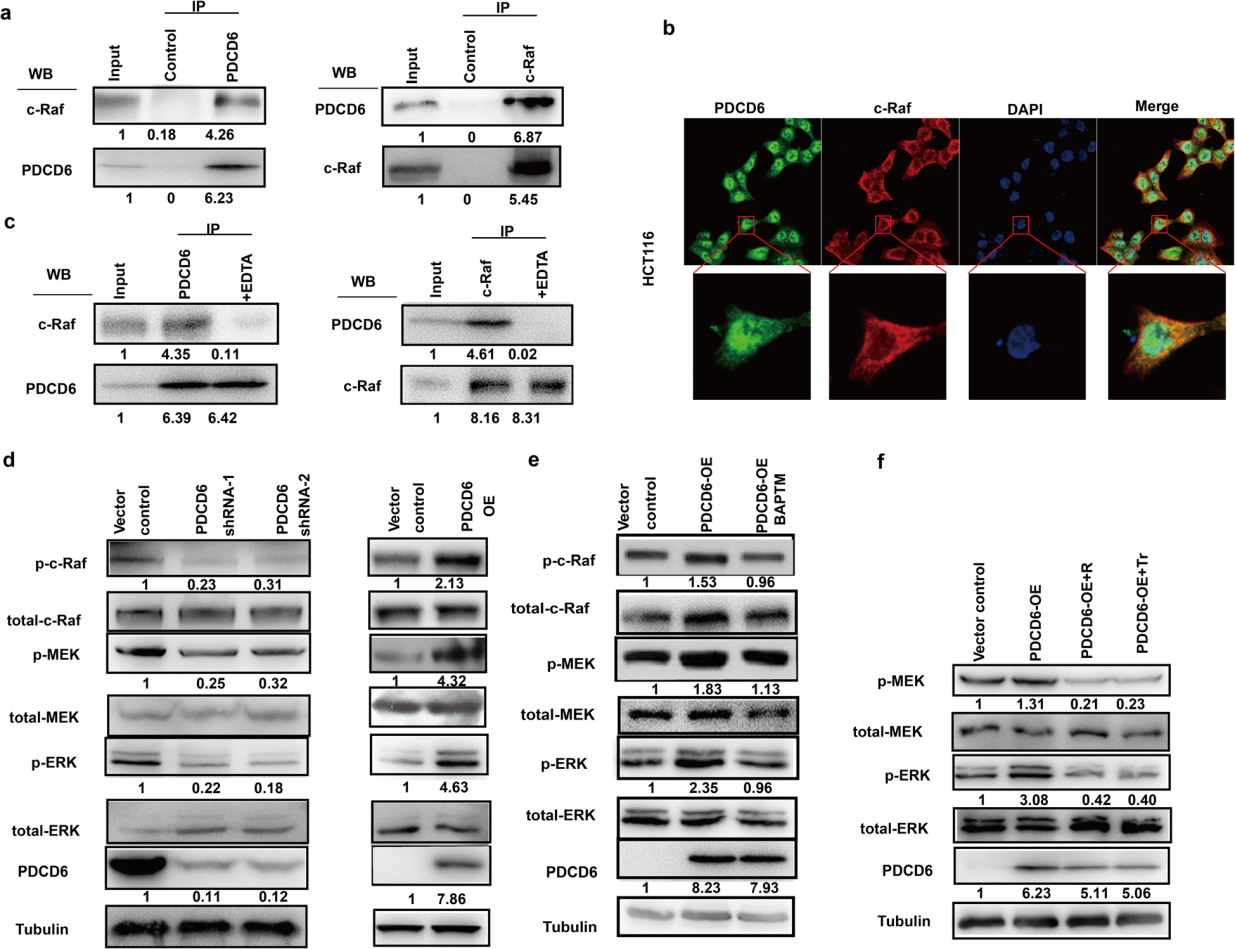
PDCD6 is physically associated with c-Raf and activates the MAPK/ERK signaling pathways in CRC cells. **a**. Coimmunoprecipitation assays using anti-PDCD6 antibody and anti-c-Raf antibodies showed that PDCD6 interacts with c-Raf in HCT116 cells. **b**. Confocal IF staining of PDCD6(green) and c-Raf (red) in HCT116 cells. The nuclei were stained by DAPI (blue). The results from one of two comparable experiments are shown. **c**. HCT116 cell lysate was immunoprecipitated with anti-PDCD6 and anti-c-Raf antibodies in the presence, and absence of EDTA. **d**. Immunoblot analysis of the expression of PDCD6 and the total and phosphorylated levels of c-Raf/MEK/ERK in HCT116 cells with PDCD6 knocked down and overexpression. Tubulin was used as a loading control. Bands were quantified with ImageJ software. **e**. Immunoblot analysis of PDCD6 the total and phosphorylated level of c-Raf/MEK/ERK in HCT116 cells overexpressing PDCD6 in the presence of the Ca^2+^ chelator BAPTM. **f**. Immunoblot analysis of PDCD6, total MEK/ERK and phosphorylated MEK/ERK in cells with PDCD6-OE, PDCD6-OE + RAF709 (R) and PDCD6-OE + Trametinib (Tr), respectively. Tubulin was used as a loading control

To further explore the underlying mechanism, we performed immunoprecipitation and mass spectrometry analyses to identify PDCD6-associated proteins. Mass spectrometry analysis identified a series of proteins in the anti-PDCD6 group. We found the c-Raf peptide, IGDFGLATVK. C-Raf (Raf-1) is a major effector recruited by the GTP-binding protein Ras that activates the MAPK pathway [34]. Furthermore, we validated this result with bidirectional co-IP followed by Western blotting of CRC cells (Fig. 5a). Additionally, immunofluorescence staining demonstrated that PDCD6 was colocalized with c-Raf in the cytoplasm of HCT116 cells (Fig. 5b). Next, we examined whether Ca^2+^ is required for the interaction between PDCD6 and c-Raf. The results showed that the addition of 5 mM EDTA could abolish the c-Raf interaction with PDCD6. Furthermore, when an anti-c-Raf antibody reciprocally coprecipitated endogenous PDCD6, EDTA abolished the interaction either (Fig. 5c). These data suggest that Ca^2+^ is indispensable for the interaction between these two proteins.

Next, we assessed the effect of PDCD6-KD and PDCD-OE on MAPK signaling pathway activity. The results showed that the phosphorylation of c-Raf/MEK/ERK was markedly decreased in the PDCD6-KD HCT116 cells compared with the control cells. In contrast, the overexpression of PDCD6 enhanced c-Raf/MEK/ERK phosphorylation levels (Fig. 5d). The cell-permeable calcium chelator BAPTM/AM was used to analyze Ca^2+^ effects on the PDCD6-mediated signaling pathway. C-Raf/MEK/ERK phosphorylation induced by PDCD6 overexpression was abolished by BAPTM/AM (Fig. 5e). Raf 709 and Trametinib are MAPK inhibitors that significantly inhibit the phosphorylation of Raf and MEK by directly blocking the function of ERK [35–38]. Immunoblotting analysis revealed that increased phosphorylation of MEK and ERK in the PDCD6-OE HCT116 cell line was significantly inhibited by RAF709 and Trametinib (Fig. 5f). Furthermore, we compared the inhibition function by PDCD6-KD and c-Raf KD, and their combination in the MAPK pathway. The results showed the inhibition effect of PDCD6-KD and c-Raf-KD was obviously, in which p- MEK and p-ERK were decreased dramatically. And the inhibition of the knockdown of PDCD6 and c-Raf in combination was not further lower than the knockdown of c-Raf, which further confirmed that c-Raf was important and necessary in PDCD6 functions in promoting cancer progression (Supplementary Fig. S2a). We combined oxaliplatin, a typical treatment used in the clinic for CRC, with downregulation of PDCD6 to test their inhibition on cell viability. The data showed that the combination downregulation of PDCD6 with oxiliplatin had a further benefit to CRC (Supplementary Fig. S2b). RAF709 and Trametinib could also inhibited cell proliferation (Supplementary Fig. S2c and S2d). These data demonstrate that PDCD6 promotes tumor growth by interacting with c-Raf and regulating the c-Raf/MEK/ERK signaling pathway.

### The positive correlation between PDCD6 and c-Raf/MEK/ERK signaling pathway in tumor tissues from xenografts and patients with CRC

**Fig. 6:**
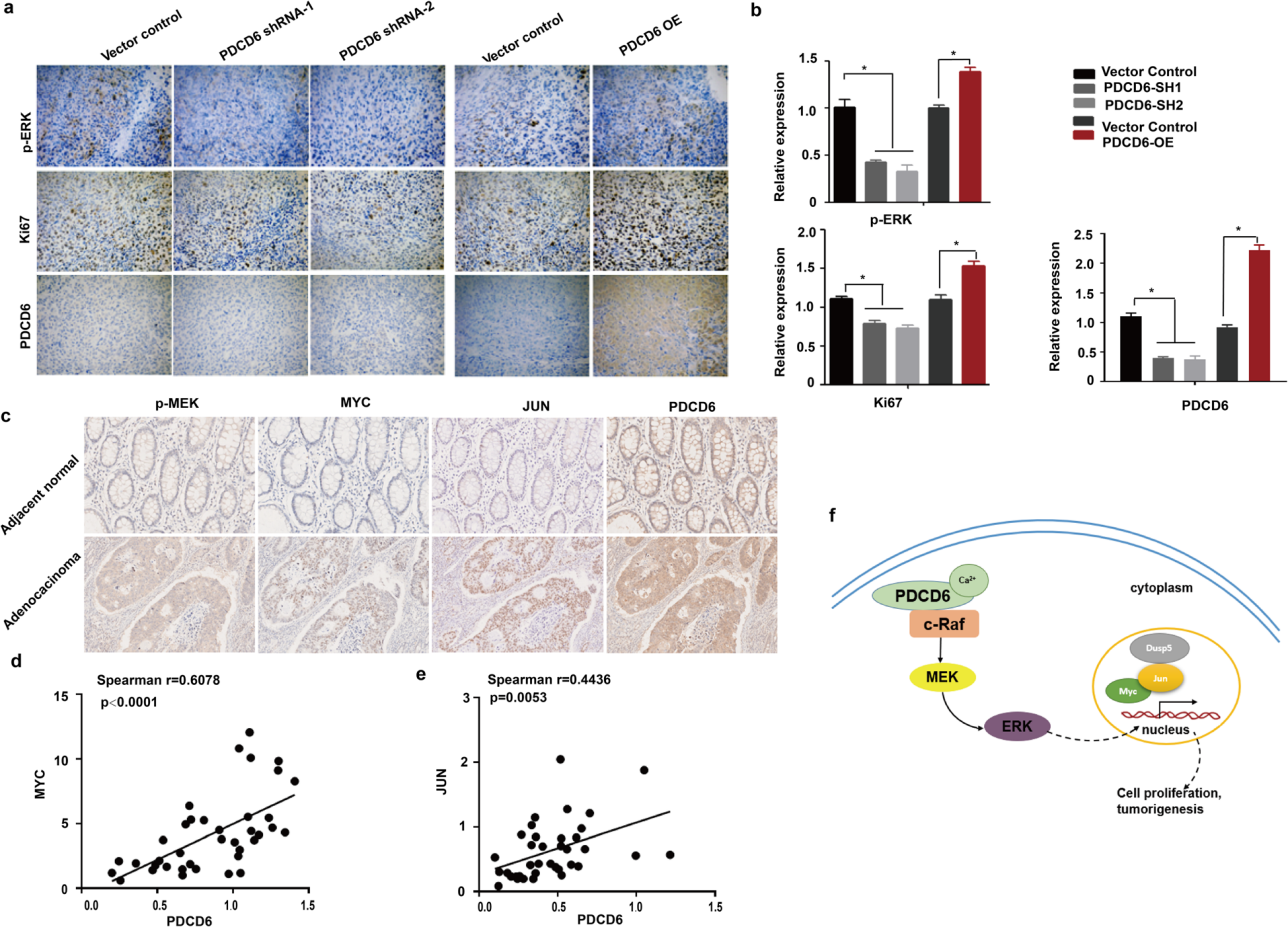
The positive correlation between PDCD6 and c-Raf/MEK/ERK signaling pathway in tumor tissues from xenografts and patients with CRC. **a**. Representative immunohistochemistry images of the expression of PDCD6, Ki67, and p-ERK in xenograft tumors from nude mice subcutaneously injected with HCT116 cells. *, *P* < 0.05. **b**. Statistical analysis of protein relative expression in A. **c**. Representative immunohistochemical staining of p-MEK, MYC, JUN, and PDCD6 from CRC tissues and adjacent normal tissues. **d** and **e**. Correlation analysis for the expression of PDCD6, MYC and JUN in 38 tissues of patients with CRC. **f**. A model showing the mechanisms of PDCD6-mediated colorectal cancer cell proliferation

We already proved that PDCD6 can influence the c-Raf/MEK/ERK signaling pathway from the cell line. Furthermore, we validated the pathway from tumor tissues from xenografts and patients with CRC. Immunohistochemical examination of the tumor xenografts revealed that the phosphorylation of c-Raf/MEK/ERK was markedly suppressed in PDCD6-KD HCT116 cells xenografts, whereas c-Raf/MEK/ERK phosphorylation was markedly enhanced in the PDCD6-OE compared with the vector control cell xenografts. We analyzed the proliferation marker Ki67 and found that the proliferation rate had the same trend as the phosphorylation of ERK under different PDCD6 expression conditions. Apoptotic marker cleaved-Caspase 3 was also assessed, which has the opposite results compared with Ki67. The phosphorylation of Stat 3, which served as a control, did not change significantly (Fig. 6a, b and Supplementary Fig. S3a). HE staining revealed apparent tumor necrosis in the downregulated of PDCD6 compared with the control group, and the upregulated showed intense positive staining with smaller cell sizes and contracted nucleus (Supplementary Fig. S3b). We next detected and analyzed the phosphorylation of p-MEK and the expression of the MAPK signal pathway downstream associated proteins to explore the correlation between PDCD6 and the MAPK pathway in colon cancer tissues. The results show that the phosphorylation of p-MEK and the expression of MYC, JUN and PDCD6 were higher in tumor tissues than in adjacent normal tissues (Fig. 6c). Furthermore, we explored the correlation between PDCD6 expression and the MAPK signal pathway downstream associated genes in tumor tissues. The results showed that the RNA expression levels of PDCD6 had a high degree of positive correlation with JUN ((Spearman’s r = 0.6078, *P* < 0.0001) and MYC (Spearman’s r = 0.4436, *P* = 0.0053) from 38 fresh CRC tissues (Fig. 6d and e). These data demonstrate that there is a significant positive correlation between PDCD6 and the c-Raf/MEK/ERK signaling pathway.

## Discussion

Previous studies have revealed that PDCD6 was involved in cancer development; The expression of PDCD6 varies in different tumors, indicating that PDCD6 plays different roles in different cancer types [21, 24]. The MAPK/ERK pathway stimulates cellular proliferation and invasion; however, its activation can also increase cellular apoptosis or antagonize pro-oncogenic input from other signals. The effect that predominates depends on the intensity of the signal and the context or tissue in which the signal is aberrantly activated [39]. The downstream MAPK/ERK signaling is predominantly activated by upstream Raf signaling. Our study found that PDCD6 promotes tumor growth by interacting with c-Raf and regulating downstream the MAPK kinase pathway. So, the opposite effect of PDCD6 might due to the opposite role of raf in different tumor types, in which the tissue-specific tumor microenvironment and the intensity of which signal activated are different.

In the present study, we explored the role of PDCD6 in the development and progression of colorectal cancer and the prognostic value of PDCD6 in patients with CRC. We found that PDCD6 was overexpression in CRC tissues. The functional experiments showed that PDCD6 depletion significantly inhibited colorectal cancer tumorigenesis and PDCD6 overexpression enhanced the proliferation and tumor growth of CRC cells. These findings and findings from clinical studies suggest the PDCD6 plays an oncogenic role in the pathogenesis of colorectal cancer. Moreover, PDCD6 overexpression tends to positively correlate with the tumor stage in patients with CRC, indicating a potential correlation between PDCD6 and malignancies. Furthermore, a Kaplan-Meier analysis revealed that PDCD6 overexpression in tumor cells had a significantly worse prognostic impact on the disease-free survival of patients with CRC, indicating that PDCD6 is a predictor of the survival of patients with colorectal cancer. The function of PDCD6 in CRC was consistent with previously reported results regarding ovarian cancer [23], and PDCD6 overexpression has been reported in hepatomas and lung cancer tissue, suggesting that this protein plays an oncogenic role in the progression of these types of tumors [21]. Overall, our clinical findings demonstrated that PDCD6 is closely associated with the progression of human CRC, and indicated that PDCD6 could serve as a useful biomarker for the prognosis of patients with CRC.

Previous studies have reported that PDCD6 promotes breast cancer growth and metastasis by regulating the cytoskeleton and mediating the proapoptotic activity of cisplatin and TNF-α through the downregulation of NF-κB expression in different biological process [22, 40]. However, by using RNA sequencing, we found that the MAPK signaling pathway is the main pathway regulated by PDCD6 in CRC. As the MAPK signaling pathways serves as a central node that regulates cell proliferation and survival [41, 42], our data indicated that PDCD6 is important for MAPK pathway activation and the growth of CRC cells. For the first time, our study reported that PDCD6 affects the MAPK signaling pathway in CRC, supplementing of the mechanistic investigation of PDCD6 in cancer. Proteomics analysis and immunoprecipitation analyses indicated that PDCD6 is physically associated with c-Raf and subsequent assays revealed that PDCD6 promotes colorectal cancer development and progression by binding c-Raf and increasing its phosphorylation level, which is consistent with previous reports on the tumor-promoting role of c-Raf [13, 43, 44], indicating that c-Raf is an executor of PDCD6 in colorectal cancer. Our results further revealed that the PDCD6/c-Raf complex activates the Raf/MEK/ERK pathway to promote the CRC progression. However, the mechanism of how the PDCD6/c-Raf complex regulates the phosphorylation levels of c-Raf requires further investigation.

Calcium ions are the secondary intracellular messenger that regulate numerous biological processes [45, 46]. Increasing evidence has suggested the role of PDCD6 as a Ca^2+^-responsive adaptor protein [47–49]. Upon binding to Ca^2+^, PDCD6 undergoes a conformational change that facilitates its interaction with various proteins [50, 51]. This conformational change enables PDCD6 to interact with various proteins [51–53]. It was experimentally verified that the interaction between PDCD6 and c-Raf requires the presence of Ca^2+^, suggesting that the interaction between PDCD6 and c-Raf is possibly due to the conformational change caused by the combination of PDCD6 and Ca^2+^, thus affecting the phosphorylation of downstream signaling pathways. Changes in the levels of intracellular Ca^2+^ provide dynamic and highly versatile signals that regulate cell proliferation [46]. The Ca^2 + −^dependence can be excluded by the fact that Ca^2+^ is essential for the effect of PDCD6 on colorectal cancer. Therefore, the effect of Ca^2+^ on cell proliferation is likely partially attributed to PDCD6.

RAF709 and Trametinib, which are effective MAPK pathway effective inhibitors, have been shown to be anticancer agents in multiple tumor types [54, 55]. To further show that PDCD6 affects the MAPK signaling pathway, RAF709 and Trametinib were used to stimulate PDCD6-OE HCT-116 cells and to observe the changes in the MAPK signaling pathway. Although PDCD6 was overexpressed, the MAPK signaling pathway was strongly inhibited after adding these two inhibitors. These results suggested that the effect of PDCD6 on CRC growth is mediated by its regulating effect on the MAPK signaling pathway. Moreover, these findings suggest the potential for antagonizing Raf/MEK/ERK signaling as a strategy to inhibit the growth of tumors hyperactivated by PDCD6.

The results from the cell lines were validated in xenografts tumor tissues to confirm their reliability. We proved that PDCD6 and c-RAF/MEK/ERK were positively correlated at the protein level by IHC in tumor tissues from xenografts and patients with CRC, which will further facilitate translational medicine research on PDCD6. In addition, this cascade mediates its function mainly through the regulation of several vital genes including MYC and JUN [28]. Consistent with the IHC analysis, the positive correlation of PDCD6 and JUN and MYC mRNA levels indicated that PDCD6 effects on CRC have a closely clinicopathological relevance.

PDCD6 has a higher expression in colorectal cancer. On one hand, PDCD6 could be used as a drug target for screening PDCD6 specific colorectal cancer treatment drug. On the other hand, PDCD6 is also a Ca^2+^ binding protein, our results show that the PDCD6 play its role to promote cancer only when it combined with Ca^2+^, so we can also inhibit Ca^2+^ as targeting PDCD6 treatment project for the colorectal cancer patients. Moreover, combination therapy of conventional drugs with PDCD6-targeted specific drugs or Ca^2+^ inhibitor drugs would be a good direction for the PDCD6-overexpressed patients. Further studies on drug exploration targeted PDCD6 could help to establish the true significance in clinical therapy. The CRC patients will benefit from the elimination of PDCD6.

## Conclusions

Based on these data, we proposed a model of the mechanism for the growth-promoting effect of PDCD6 (Fig. 6f). In this model, PDCD6 may undergo a conformational change by binding with Ca^2+^ that facilitates its interaction with c-Raf in the cell cytoplasm. The stabilized PDCD6/c-Raf complex subsequently activates the Raf/MEK/ERK signaling pathway and regulates the downstream transcription factors including MYC and JUN, which resulting in colorectal cancer growth and progression. In summary, our study provided mechanistic evidence of the involvement of PDCD6 in the regulation of CRC cell growth. This study suggested that PDCD6 could be a potential prognostic biomarker and therapeutic target for CRC, and further investigation is warranted to achieve its clinical application.

## Supplementary information

**Additional file 1: Figure S1.** The PDCD6 expression in different patient samples from the GEPIA database of different kinds of diseases. **Figure S2a.** PDCD6-KD and c-Raf-KD inhibited the MAPK pathway. **Figure S2b.** Combined downregulation of PDCD6 with oxiliplatin effect on cell proliferation in HCT116 and HCT15. **Figure S2c.** Cell proliferation assays on RAF709 and Trametinib treatment. **Figure S2d.** Colony formation assays on RAF709 and Trametinib treatment. **Figure S3a.** Representative immunohistochemistry images of the expression of p-c-Raf, p-MEK, c-Caspase 3 and p-Stat 3 in xenograft tumors from nude mice subcutaneously injected with HCT116 cells. *, *P* < 0.05. **Figure S3b.** HE staining showed the influences of PDCD6-KD and PDCD6-OE on mice tumor samples. **Table S1.** Characteristics of 93 patients with colorectal cancer included in the study. **Table S2.** For the knockdown of human PDCD6-specific shRNAs. **Table S3.** For the knockdown of human PDCD6-siRNA and c-Raf-siRNA. **Table S4.** Oligonucleotide sequences used in reverse-transcription PCR and real-time PCR.

## Data Availability

The datasets used and/or analyzed during the current study are available from the corresponding author on reasonable request.

## Abbreviations

CRC: Colorectal cancer
RNA-seq: RNA-sequence
MS: Mass Spectrum
Co-IP: Co-Immunoprecipitation
IHC: Immunohistochemistry
HE: Hematoxylin-eosin
qRT-PCR: Quantitative real-time PCR
MAPK: Mitogen-activated protein kinase
PDCD6: Programmed cell death protein 6
ALG-2: Apoptosis-linked gene-2
FBS: Fetal bovine serum
ACC: Adrenocortical carcinoma
BLCA: Bladder Urothelial Carcinoma
BRCA: Breast invasive carcinoma
CESC: Cervical squamous cell carcinoma and endocervical adenocarcinoma
CHOL: Cholangiocarcinoma
COAD: Colon adenocarcinoma
DLBC: Lymphoid Neoplasm Diffuse Large B-cell Lymphoma
ESCA: Esophageal carcinoma
GBM: Glioblastoma multiforme
HNSC: Head and Neck squamous cell carcinoma
KICH: Kidney Chromophobe
KIRC: Kidney renal clear cell carcinoma
KIRP: Kidney renal papillary cell carcinoma
LAML: Acute Myeloid Leukemia
LGG: Brain Lower Grade Glioma
LIHC: Liver hepatocellular carcinoma
LUAD: Lung adenocarcinoma
LUSC: Lung squamous cell carcinoma
OV: Ovarian serous cystadenocarcinoma
PAAD: Pancreatic adenocarcinoma
PCPG: Pheochromocytoma and Paraganglioma
PRAD: Prostate adenocarcinoma
READ: Rectum adenocarcinoma Esophageal carcinoma
SARC: Sarcoma
SKCM: Skin Cutaneous Melanoma
STAD: Stomach adenocarcinoma
TGCT: Testicular Germ Cell Tumors
THCA: Thyroid carcinoma
THYM: Thymoma
UCEC: Uterine Corpus Endometrial Carcinoma
UCS: Uterine Carcinosarcoma
pTNM: Tumor-node-metastasis
pT: Pathological tumor
pN: Pathological node
DFS: Disease-free survival
GO: Gene ontology
KEGG: Kyoto Encyclopedia of Genes and Genomes
GSEA: Gene set enrichment analysis
