# Supplementary material for: PDCD6 cooperates with C-Raf to facilitate colorectal cancer progression via Raf/MEK/ERK activation
Source: J Exp Clin Cancer Res. 2020 Aug 3;39:147. doi: 10.1186/s13046-020-01632-9 (PMC7398064; doi:10.1186/s13046-020-01632-9)
Supplement: Supplementary file 1 — Additional file 1: Figure S1. The PDCD6 expression in different patient samples from the GEPIA database of different kinds of diseases. Figure S2a. PDCD6-KD and c-Raf-KD inhibited the MAPK pathway. Figure S2b. Combined downregulation of PDCD6 with oxiliplatin effect on cell proliferation in HCT116 and HCT15. Figure S2c. Cell proliferation assays on RAF709 and Trametinib treatment. Figure S2d. Colony formation assays on RAF709 and Trametinib treatment. Figure S3a. Representative immunohistochemistry images of the expression of p-c-Raf, p-MEK, c-Caspase 3 and p-Stat 3 in xenograft tumors from nude mice subcutaneously injected with HCT116 cells. *, P < 0.05. Figure S3b. HE staining showed the influences of PDCD6-KD and PDCD6-OE on mice tumor samples. Table S1. Characteristics of 93 patients with colorectal cancer included in the study. Table S2. For the knockdown of human PDCD6-specific shRNAs. Table S3. For the knockdown of human PDCD6-siRNA and c-Raf-siRNA. Table S4. Oligonucleotide sequences used in reverse-transcription PCR and real-time PCR. [file 13046_2020_1632_MOESM1_ESM.docx]

**Additional file**

**Figure S1**

**
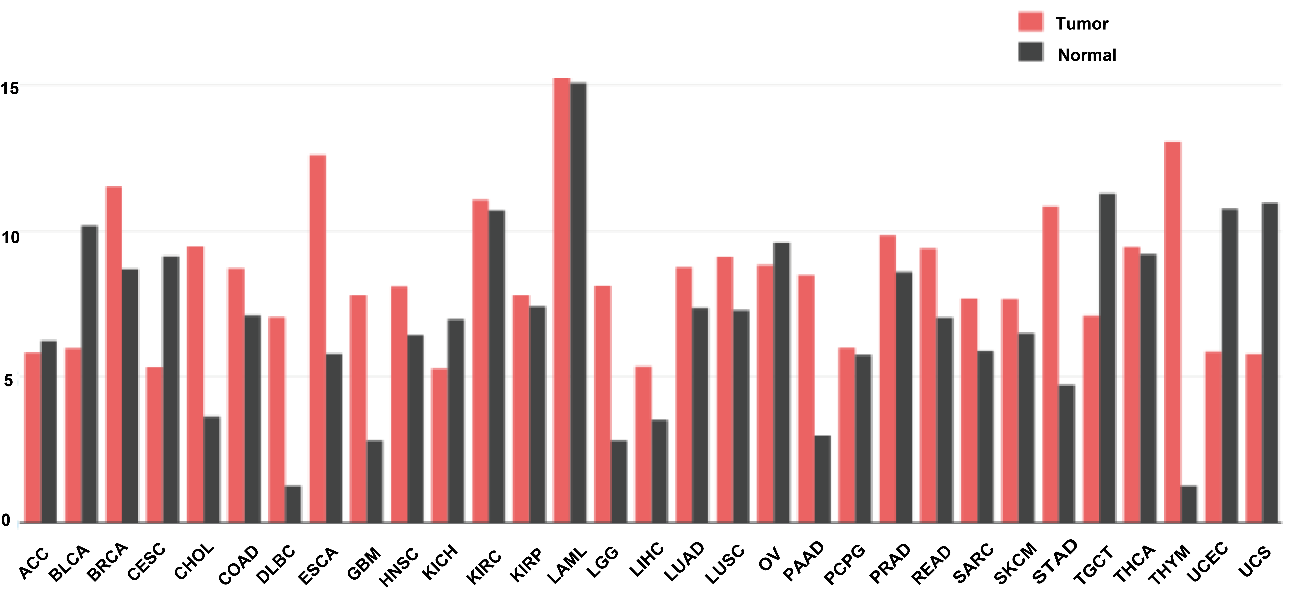
**

**Figure S1.** The PDCD6 expression in different patient samples from the GEPIA database of different kinds of diseases.

**Figure S2**

**
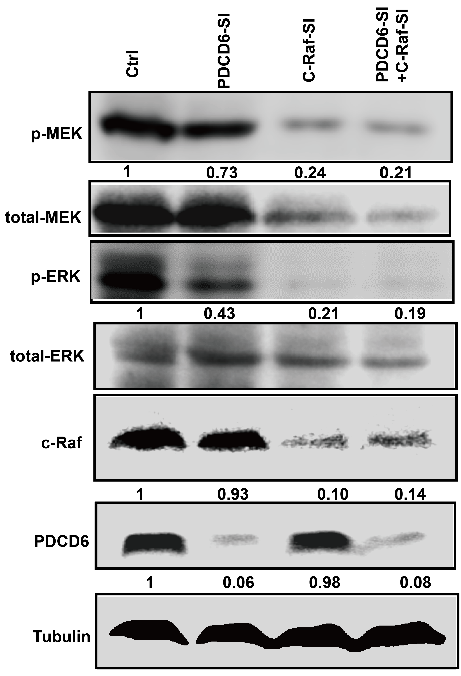
**

**Figure S2a.** PDCD6-KD and c-Raf-KD inhibited the MAPK pathway.

**
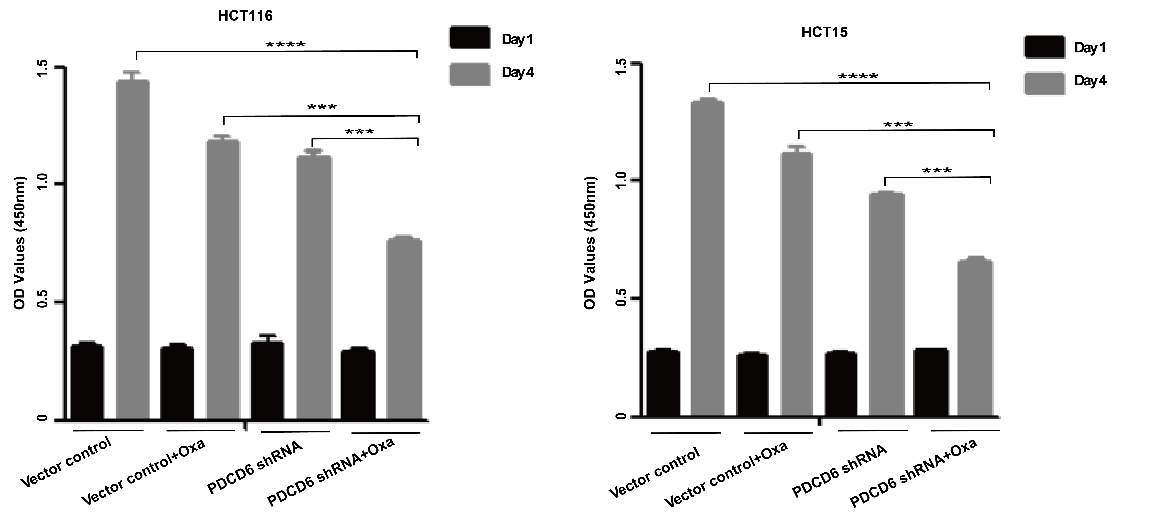
**

**Figure S2b.** Combined downregulation of PDCD6 with oxiliplatin effect on cell proliferation in HCT116 and HCT15.


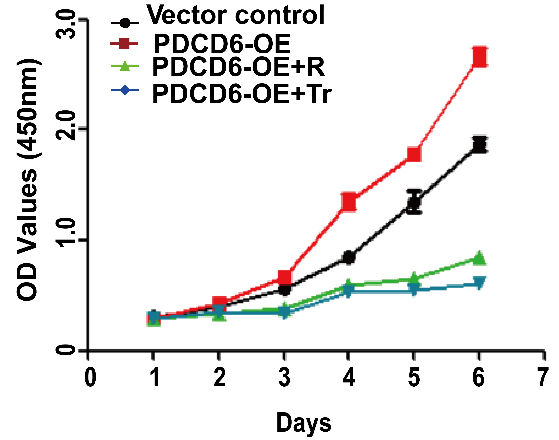


**Figure S2c.** Cell proliferation assays on RAF709 and Trametinib treatment.


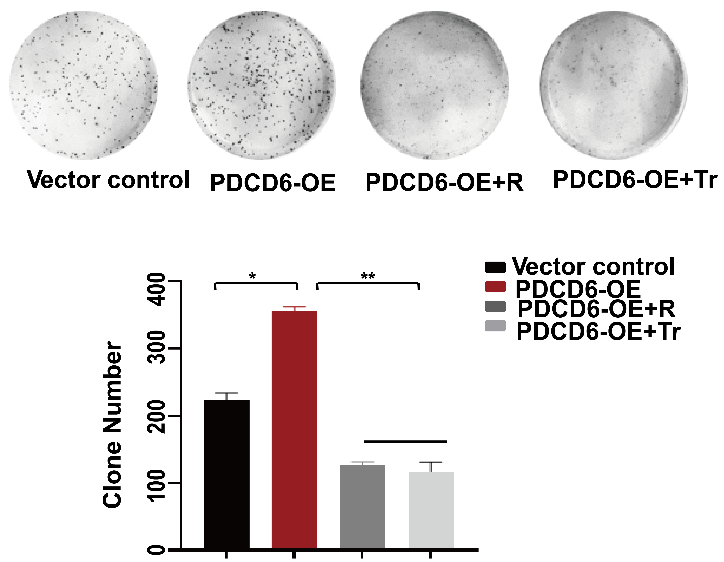


**Figure S2d.** Colony formation assays on RAF709 and Trametinib treatment.

**Figure S3**

**
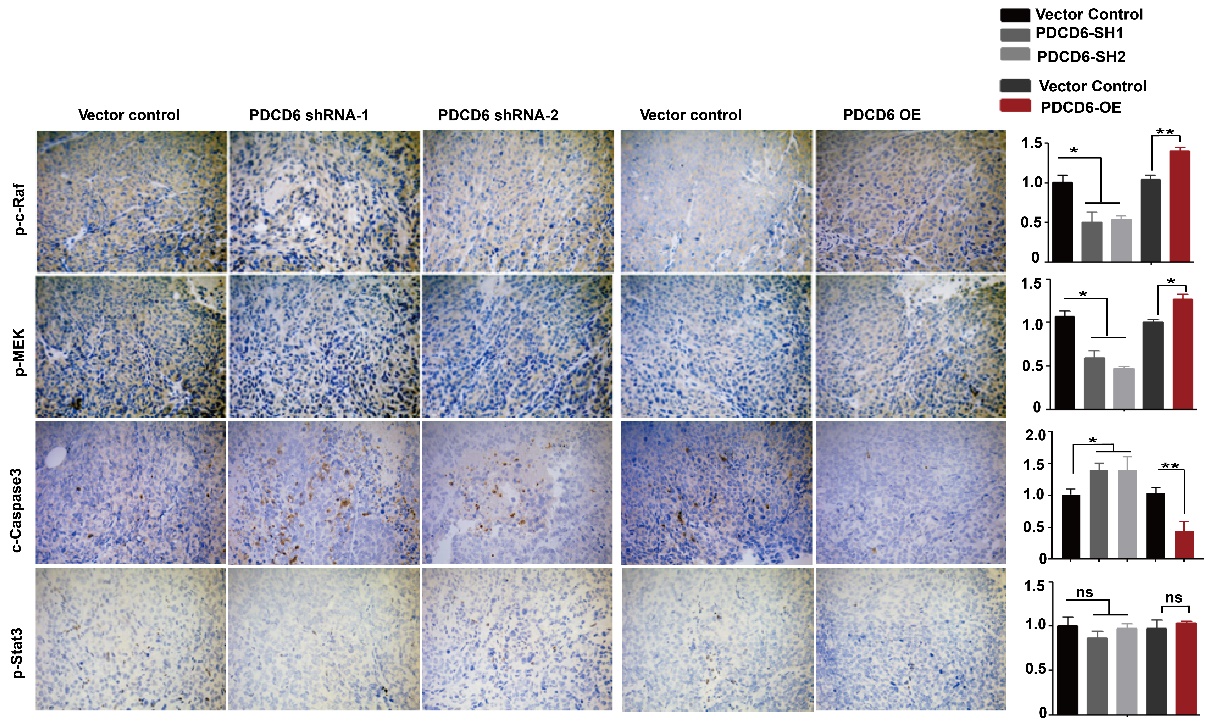
**

**Figure S3a.** Representative immunohistochemistry images of the expression of p-c-Raf, p-MEK, c-Caspase 3 and p-Stat 3 in xenograft tumors from nude mice subcutaneously injected with HCT116 cells. *, P < 0.05.

**
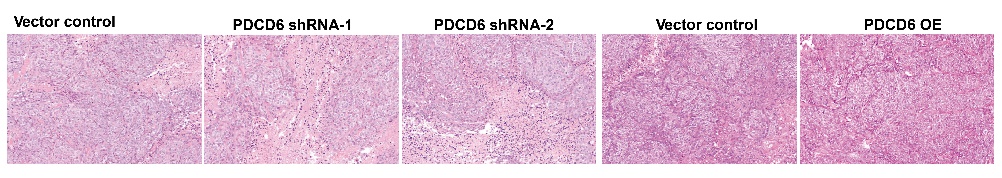
**

**Figure S3b.** HE staining showed the influences of PDCD6-KD and PDCD6-OE on mice tumor samples.

**Table S1. Characteristics of 93 patients with colorectal cancer included in the study.**

**Characteristics No. of patients (N=93)**

Gender

Male 49

Female 44

Age

≤60 33

>60) 60

Tumor size (cm)

<5 41

≥5 52

Stage

I 3

II 23

III 65

IV 3

Location

Colon 53

[Rectum](javascript:;) 40

Distance metastasis

Positive 2

Negative 91

**Table S2. For the knockdown of human PDCD6-specific shRNAs**

**No. Sequence**

shRNA1 CCGGGCAGAGGTTGACGGATATATTCTCGAGAATATATC

CGTCAACCTCTGCTTTTTG

shRNA2 CCGGTAGTAGCTGTATCGTTCTAATCTCGAGATTAGAAC

GATACAGCTACTATTTTTG

**Table S3. For the knockdown of human PDCD6-siRNA and c-Raf-siRNA**

**No. Sequence**

si-PDCD6_1 CCATGTTTGACCGTGAGAAs

si-PDCD6_2 GCAGAGGTTGACGGATATA

si-PDCD6_3 GCTGGATTCAGGTGTCGTA

si-c-Raf_1 GCACCAAAGTACCTACTAT

si-c-Raf_2 GCAGCAGCCTCTACAAACA

si-c-Raf_3 GCACTACCTTCTTTGACTA

**Table S4. Oligonucleotide sequences used in reverse-transcription PCR and real-time PCR.**

| **NO**. | \| **Gene** \| \| --- \| | **Forward (5’to 3’)** | **Backward (5’to 3’)** |
| --- | --- | --- | --- | --- |
| 1. | PDCD6 | ATGGCCGCCTACTCTTACC | TCCTGTCTTTATCGACCCTCTG |
| 2 | Actin | CATGTACGTTGCTATCCAGGC | CTCCTTAATGTCACGCACGAT |
| 3 | MYC | GTCAAGAGGCGAACACACAAC | TTGGACGGACAGGATGTATGC |
| 4 | DUSP5 | ACAGCCCTGCTGAATGTCTC | GGAGCTAATGTCAGCCGTGT |
| 5 | JUN | TCCAAGTGCCGAAAAAGGAAG | CGAGTTCTGAGCTTTCAAGGT |
| 6 | CACNB4 | GCAAAAAGTGACGGAGCACA | TAACCTTTCAGTGACGGCCC |
| 7 | FGFR4 | ACATCATCCTGTACGCGTCG | GGAGAGCTTCTGCACAGTGG |
| 8 | GADD45B | TACGAGTCGGCCAAGTTGATG | GGATGAGCGTGAAGTGGATTT |
| 9 | PDGFA | ATACCTCGCCCATGTTCTGG | ATGCTGTGGATCTGACTGCG |
| 10 | DUSP10 | TGAAGCACACTCGGATGACC | CCTCGAACTCTAGCAACTGCC |
| 11 | PTRRR | ACCTATCGCCCATCACATTACA | GCGGTGGTAGCTTTGATCTCA |
| 12 | DUSP2 | TGCCCCAACCACTTTGAGG | AGTCAATGAAGCCTATGGCCT |
| 13 | CACNA1G | ACACTTGGAACCGGCTTGAC | AGCACACGGACTGTCCTGA |
| 14 | CACNA1H | ATGCTGGTAATCATGCTCAACTG | AAAAGGCGAAAATGAAGGCGT |
| 15 | NTRK2 | ACCCGAAACAAACTGACGAGT | AGCATGTAAATGGATTGCCCA |
| 16 | PTPN7 | CTACATCCGAGGCTATGACGG | GGCCAGTAGTGGACACATTTC |
| 17 | NFATC1 | CACCCAAGAGCCGACCTG | CAGTCGGAGACTCGTCCCT |
| 18 | RASGRF2 | CAGAAGAGCGTGCGCTACAA | TGCTCGCCCTCGAAGTAGAA |
| 19 | RASGRF1 | AGCTTGTGTCATCTGAGGGC | ATCCCCAGGTAAGGGACACA |
| 20 | PLA2G4A | ATGGATGAAACTCTAGGGACAGC | CTGGGCATGAGCAAACTTCAA |
| 21 | MECOM | TATCCACGAAGAACGGCAATATC | CATGGAAACTTTTGGTGATCTGC |
